# Paclitaxel-induced acute myocardial infarction: a case report and literature review

**DOI:** 10.1186/s12872-024-03814-1

**Published:** 2024-03-19

**Authors:** Gi Eun Kim, Ayman R. Ibrahim, Duha Shalatouni, Nadin H. Abouzeid, Fahmi Othman

**Affiliations:** 1 Department of Internal Medicine, Hamad General Hospital, Hamad Medical Corporation, Doha, Qatar; 2 Department of Cardiology, Heart Hospital, Hamad Medical Corporation, Doha, Qatar

**Keywords:** Paclitaxel, Acute myocardial infarction, Chemotherapy, Case report

## Abstract

**Background:**

Paclitaxel is a chemotherapeutic agent commonly used for ovarian, lung, breast carcinoma, and Kaposi’s sarcoma. Its common side effects include hypersensitivity reaction, bone marrow suppression, and peripheral neuropathy. However, a rare and life-threatening side effect is paclitaxel-induced myocardial infarction.

**Case presentation:**

A 71-year-old man with type 2 diabetes mellitus, hypertension, heavy smoking history, previous coronary artery disease with percutaneous coronary intervention (PCI) in left anterior descending artery (LAD), and non-small lung cancer presented with non-ST elevation myocardial infarction during infusion of paclitaxel infusion. Coronary angiogram showed de novo three vessel disease with 70% stenosis in ostial to distal left main artery (LM) and 80% in-stent re-stenosis in proximal to mid left anterior descending artery.

**Conclusions:**

Physicians should be keeping this in mind when dealing with patients on paclitaxel, especially if they have previous risk factors for coronary artery disease.

**Supplementary Information:**

The online version contains supplementary material available at 10.1186/s12872-024-03814-1.

## Background

The chronic inflammation in cancer predisposes patients to arterial and venous thromboembolism, including myocardial infarction [1]. Not only that, but chemotherapy itself is increasingly associated with cardiotoxicity with reported cases of myocardial infarction induced by various chemotherapeutic agents. Potential inducers of coronary vasospasm are 5-fluorouracil, capecitabine, paclitaxel, gemcitabine, rituximab and sorafenib. Other chemotherapeutic drugs are thought to cause direct toxicity on endothelial cells and cause erosion with atherosclerotic plaque rupture, such as cisplatin and vinca alkaloids [1]. We present a case of a patient with metastatic non-small lung cancer (cT4N2M1) who developed acute coronary syndrome during infusion of paclitaxel infusion.

## Case presentation

A 71-year-old man with metastatic non-small cell lung carcinoma and cardiovascular risk factors of type 2 diabetes mellitus, hypertension, heavy smoking history of 80 pack years, and previous coronary artery disease, presented to oncology unit for cycle 8 of pembrolizumab and cycle 5 of paclitaxel and carboplatin. He had coronary angiogram in 2014 which showed distal LM 20–30%, left circumflex artery (LCX) 80%, right coronary artery (RCA) chronic total occlusion, and PCI was done to LAD. He has no family history of coronary artery disease. He was recently diagnosed with poorly differentiated squamous cell carcinoma with staging of cT4N2M1. He had previously progressed on four cycles of pembrolizumab, thus carboplatin and paclitaxel were added two months prior to our event. Patient received a total of 7 doses of pembrolizumab and 4 doses of carboplatin and paclitaxel with no events.

**Fig. 1:**
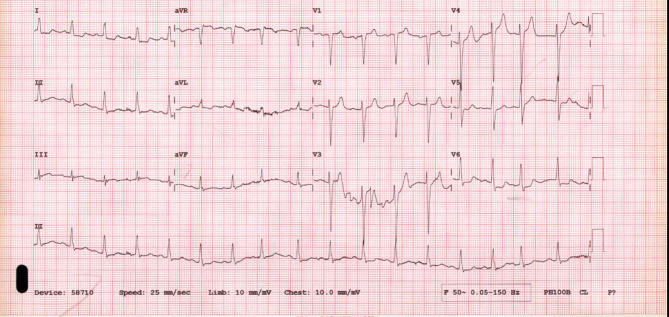
Initial electrocardiogram

During the eighth session of chemotherapy, pre-medications including diphenhydramine 50 mg, dexamethasone 20 mg, and netupitant were given, and patient completed the pembrolizumab infusion without any complications. However, after starting paclitaxel infusion, the patient developed sudden onset left side chest pain, radiating to left arm associated with shortness of breath. On examination, he was alert and oriented, he was tachypneic (respiratory rate of 25 breathes per minute), tachycardic (heart rate of 109 beats per minute), normotensive (blood pressure of 123/58mmHg), and he was maintaining his oxygen saturation (oxygen saturation 93%) on 4 L of oxygen. Physical examination was remarkable for raised jugular venous pressure with diffuse wheezes and bilateral basal crackles on chest. Paclitaxel was immediately stopped, and the patient received hydrocortisone and diphenhydramine as possible hypersensitivity reaction. Electrocardiogram was showing normal sinus rhythm, ST depression 1 mm in the inferolateral leads and 1 mm ST elevation in aVR (Fig. 1). Laboratory results revealed troponin T levels of 32 ng/L, 74 ng/L, then 1439 ng/L. The peak troponin level was 2834 ng/L (normal value 3–15 ng/L). He was diagnosed with non-ST elevation myocardial infarction and shifted to coronary care unit.

He received full anti-ischemic medications including his home medications which were aspirin 100 mg, bisoprolol 2.5 mg, isosorbide dinitrate 20 mg, rosuvastatin 20 mg, and in addition, he was loaded with 300 mg clopidogrel and started on therapeutic enoxaparin.

CT pulmonary angiogram showed no evidence of pulmonary embolism. Transthoracic echocardiography showed left ventricular ejection fraction (LVEF) of 30% (compared to LVEF 49% in previous echocardiogram), with regional wall motion abnormalities in inferior and anterolateral walls (additional file 1).

Coronary angiogram was done (additional file 2) which showed 70% stenosis in ostial and distal LM, 80% stenosis in-stent restenosis in proximal to mid LAD, 80% stenosis in distal left circumflex artery, 100% chronic total obstruction in proximal right coronary artery. A drug-eluting stent (Xience Sierra 3.5 × 33 mm) was placed in the ostial LM to proximal LAD.

Unfortunately, two days after the coronary angiogram, patient developed melena with 4 gram drop in hemoglobin, for which he required two units of packed red blood cells. Upper and lower endoscopy showed only two small, flat angiodysplasia lesions oozing blood in cecum which were clipped, which stopped the oozing. Even after gastrointestinal bleeding, he was kept on dual antiplatelet, and his recovery afterwards was unremarkable, with no more drops in hemoglobin.

After several days of observation, he was discharged home with education regarding smoking cessation and lifestyle modification, in addition to dual anti-platelet therapy with aspirin and ticagrelor for one year, then with ticagrelor monotherapy lifelong. In a follow up with cardiology clinic 2 weeks after discharge, patient was well and compliant to medications.

## Discussion and conclusions

Paclitaxel is a chemotherapeutic agent in class called taxane approved by Food and Drug Administration (FDA) for ovarian, lung, breast carcinoma, and Kaposi’s sarcoma [2]. It causes cell cycle inhibition by stabilizing microtubule and activates cell apoptosis pathway. Its most common side effects include hypersensitivity reaction, bone marrow suppression, and peripheral neuropathy. Cardiovascular abnormalities are rare (< 1%) however includes bradycardia, atrial and ventricular arrhythmias, negative inotropic effect, and congestive heart failure [2]. A concerning cardiovascular side effect is life-threatening myocardial infarction, which has been reported in literature in 10 case reports [3–12].

There are various proposed mechanisms for how paclitaxel induces myocardial infarction [12]. The most proposed mechanism is acute myocardial infarction due to prolonged coronary artery vasospasm, whether it is histamine induced from castor oil used in suspension, increased intracellular calcium concentration, or allergy to paclitaxel [12].

We searched on Pubmed and Google scolar database for paclitaxel-induced myocardial infarction published till 2 October 2023 using keywords “paclitaxel”, “myocardial infarction”, “acute coronary syndrome”. There were 10 cases which are summarized in Table 1. Most of the acute myocardial infarctions occurred during or right after paclitaxel infusion (Table 1),

**Table 1:** Literature search of paclitaxel-induced myocardial infarction

Case No.	Author	Year	Age	Gender	Cardiovascular risk factors	Symptoms	Cycle of chemotherapy	Timing of onset	ECG	Coronary angiogram	Management	Outcome	Other chemotherapy
1	Hekmat	1996	67	F	None	Chest pain, dyspnea	Second	15 h after start of infusion	Inferior STEMI	Not done	Medical management	Death after 13 h after stopping infusion	None
2	Laher	1997	61	F	None	Chest pain, dyspnea	First	6 h after completion of infusion	Anterior STEMI	Not done	Medical management	Stabilized, death from non-cardiac cause	None
3	Schrader	2005	58	F	None	Chest pain, nausea	First	20 min after start of infusion	Inferior STEMI	Not done	Medical management	Alive	None
4	Gemici	2009	51	F	Recent MI s/p PCI	Chest pain, sweating	Second	Minutes after start of infusion	Anterior/inferior STEMI	80% LCx	Stent placement (BMS)	Alive	None
5	Londley	2009	48	F	None	Circulatory collapse	Fifth	After completion of infusion	Anterior/lateral STEMI	Not done	CPR, medical management	Alive	Cisplatin (previous cycle)
6	Park	2009	63	F	Hypertension	Typical angina	First	Day after completion of infusion	Anterior STEMI	LM + dLAD	Balloon angioplasty	Alive	None
7	Shah	2012	45	F	None	Chest pain, dyspnea, sweating	First	After completion of infusion	NSTEMI	Not done	Medical management	Alive (palliative care)	None
8	Esher	2014	47	F	None	Facial flushing, chest pain	Second	5 min of start of infusion	Anterior STEMI	pLAD + vasospasm in m-/d-LAD	2 DES	Death due to PEA arrest	None
9	Rawal	2016	63	M	None	Chest pain, sweating, dyspnea, hypotension	First	Just after completion of infusion	Inferior STEMI	100% RCA	PCI	Alive	None
10	Higami	2022	48	F	None	Sweating, decreased level of consciousness	First	8–10 min of start of infusion	ST elevation in I, II, III, aVL, aVF, V4-V6	Normal	None	Alive	None

In addition, most cases showed ST elevation in electrocardiogram. Coronary angiogram was done in only 5 cases, one of which showed normal coronaries suggesting only vasospasm, while 4 cases showed coronary artery stenosis, suggesting the possibility of paclitaxel not only inducing vasospasm, but also coronary artery stenosis. The compounding factor in determining that paclitaxel is causing the stenosis itself is that malignancy itself is a risk factor for CAD, and although 8 out of 10 case reports did not have any other cardiovascular risk factors (Table 1), our patient has multiple cardiovascular risk factors including type 2 diabetes mellitus, hypertension, previous CAD and stent, heavy smoking history, as well as malignancy itself.

Carboplatin is an alkylating agent that interacts with purine bases in DNA interfering with normal transcription and DNA replication and causes cancer cell apoptosis [13]. Cisplatin, which is in the same class, is known to increase risk of thromboembolism, with several case reports on cisplatin-induced myocardial infarction [14–16]. There was one retrospective cohort study that showed that there is no significant difference between cisplatin and carboplatin in risk of thromboembolism, and 15.2% of thromboembolic events from carboplatin group were arterial compared to 0% in cisplatin group, which included pulmonary embolism, cerebrovascular accidents, and myocardial infarction [13]. Thus, as our patient was on regimen including carboplatin, although he did not receive it on the day of event, it is likely an additional risk factor for thrombosis.

On the day of the event, the patient received pembrolizumab as well, which completed before MI occurred. There are no reports that linked pembrolizumab to myocardial infarction so far. Thus, we thought it most likely that paclitaxel was the culprit.

A seemingly contradictory aspect of this paclitaxel-induced adverse effect is that paclitaxel-coated balloons and stents are commonly used for coronary revascularization [17]. One possible explanation is that the adverse effect is due to coronary vasospasm from suspension medium, rather than drug itself [12]. Paclitaxel is highly lipophilic, and it requires a suspension medium when given intravenously for chemotherapy, most commonly co-solvent of ethanol and Cremophor EL™ (a polyoxyethylated castor oil) [18]. This is not necessary in drug eluting stents or balloon as they are directly applied to walls of coronary arteries. However, further studies are required to delineate the exact mechanism of paclitaxel-induced myocardial infarction.

The strength of this case report includes the high probability of adverse reaction being caused by paclitaxel, due to timing of the adverse effect right after the paclitaxel infusion, supported by other case reports describing a similar timeline. A weakness is that our patient had multiple other cardiovascular risk factors, which is a compounding factor, however other case reports also reported the presence of other cardiovascular risk factors.

Although most of the cases occurred after first or second dose of paclitaxel, one case report had the adverse effect after the fifth dose (Table 1). Therefore, it seems the adverse effect occurs more commonly after the first two doses, but it can still occur in subsequent doses.

Paclitaxel-induced myocardial infarction is a rare but fatal complication, especially in patients with previous risk factors for coronary artery disease. Physicians should be more aware of this side effect for prompt diagnosis and treatment, to prevent significant morbidity and mortality.

### Electronic supplementary material

Below is the link to the electronic supplementary material.

Supplementary Material 1

Supplementary Material 2

Supplementary Material 3

Supplementary Material 4

Supplementary Material 5

Supplementary Material 6

## Data Availability

The datasets used and/or analyzed during the current study available from the corresponding author on reasonable request.

## Declarations

### Author’s information

GK, AI, DS, NA are medical residents in internal medicine residency program at Hamad Medical Corporation. FO is consultant in cardiology at Hamad Medical Corporation.

## Abbreviations

LAD: Left anterior descending artery
LM: Left main artery
LCX: Left circumflex artery
RCA: Right coronary artery
LVEF: Left ventricular ejection fraction
PCI: Percutaneous coronary intervention
