# Supplementary material for: Paclitaxel-induced acute myocardial infarction: a case report and literature review
Source: BMC Cardiovasc Disord. 2024 Mar 19;24:167. doi: 10.1186/s12872-024-03814-1 (PMC10949626; doi:10.1186/s12872-024-03814-1)
Supplement: Supplementary file 1 — Supplementary Material 1 [file 12872_2024_3814_MOESM1_ESM.docx]

Additional file 1: Echocardiography (a) apical 4 chamber view (b) apical 2 chamber view showing severely reduced systolic left ventricular function and regional wall motion abnormalities in apex and inferior and anterolateral walls

Additional file 2: Coronary angiogram (a) left coronary angiogram in RAO caudal view showing 70% ostial and distal LM stenosis, 80% in-stent restenosis in proximal edge of proximal LAD, and 80% distal left circumflex artery stenosis (b) right coronary angiogram in RAO view showing 100% chronic total obstruction of proximal RCA (c) left coronary angiogram after stent placement in ostial LM to proximal LAD showing TIMI 3 flow
